# Complementary feeding practices for children aged 6–23 months in early childhood education institutions in urban China: A cross-sectional study

**DOI:** 10.7189/jogh.14.04043

**Published:** 2024-03-08

**Authors:** Qiong Wu, Na Meng, Xiaotong Wang, Lin Li, Jian Zhang, Yiwen Huang, Yanfeng Zhang

**Affiliations:** 1 Department of Integrated Early Childhood Development, Beijing Municipal Key Laboratory of Child Development and Nutriomics, Capital Institute of Pediatrics, Beijing, China; 2 Beijing KidsHome Children Development Centre, Beijing, China; 3 Child Healthcare Center, Children’s Hospital, Capital Institute of Pediatrics, Beijing, China

## Abstract

**Background:**

Appropriate infant and young child feeding (IYCF) plays a crucial role in promoting the healthy growth of children. Currently, many Chinese urban parents are seeking care of children from the early childhood education (ECE) institutions, however, little is known about the feeding practices of infants and young children in ECE institutions. This study aims to investigate the complementary feeding practices for Chinese urban children aged 6–23 months in ECE institutions and explore potential factors influencing their feeding practices.

**Methods:**

This cross-sectional study was conducted among primary caregivers of children aged 6–23 months in ECE institutions across 31 provinces in China from 1 March to 30 April 2023. Convenience sampling was used to recruit caregivers from centres of Gymboree Play & Music (an ECE institution). Self-administered questionnaires were designed using the online survey tool Sojump and distributed through WeChat platform, which collected information on 1) children's complementary feeding practices; 2) food frequency of seven food groups; 3) caregivers' feeding knowledge and practices; 4) frequency of children attended ECE classes and the primary caregivers' daily nurturing care time; 5) source of information on complementary feeding.

**Results:**

A total of 2731 children and their caregivers were surveyed, with 416 children aged 6–11 months and 2315 children aged 12–23 months. The prevalence of minimum dietary diversity (MDD), minimum meal frequency (MMF), and minimum acceptable diet (MAD) was 59.4, 60.6, and 39.2%, respectively. Only 17.3% caregivers believed that continued breastfeeding should be at two years or above, and 29.5% children were continuously breastfed at 12–23 months (CBF). The proportion of non-responsive feeding practices among caregivers ranged from 28 to 64%. Except for CBF, the prevalence of other key complementary feeding practices was higher among children who attending ECE classes than those not attending ECE classes (all *P* < 0.05). Moreover, children aged 12–23 months who received long-nursing care time (≥4h/d) had significantly higher MMF and MAD prevalence than those in short-nursing care time group (MMF = 66.2 vs. 58.8%, *P* = 0.0003; MAD = 44.2 vs. 38.3%, *P* = 0.0047).

**Conclusions:**

The complementary feeding practices of children aged 6–23 months in ECE institutions in urban China remained suboptimal, and non-responsive feeding practices among caregivers were common. The attendance of ECE classes and the caregivers’ daily nurturing care time could be beneficial in ensuring children to comply with complementary feeding recommendations.

Complementary feeding, generally from age 6–23 months, is crucial for children to learn to accept a wide range of foods and establish long-term dietary patterns [1]. It is well recognised that the complementary feeding period is also a vulnerable period in the lives of children as it coincides with the peak period for risk of growth faltering and nutrient deficiencies [1]. Therefore, appropriate complementary feeding is essential to foster healthy growth of children, which could reduce the risk of undernutrition and overnutrition with added long-term physical and psychological health benefits [2].

The World Health Organization (WHO) and the United Nations Children's Fund (UNICEF) jointly developed global strategy and guidelines to promote appropriate infant and young child feeding (IYCF) [3-5], along with standard indicators to monitor and guide the feeding practices of young children [6]. Globally, complementary feeding practices are generally suboptimal, with only 28.2, 50.3, and 15.9% of children worldwide met minimum dietary diversity (MDD), minimum meal frequency (MMF), and minimum acceptable diet (MAD) respectively in 2017 [7]. National data in China showed that the prevalence of MDD, MMF and MAD was 60.6, 72.4, and 43.4%, respectively [8].

Caregivers’ feeding practices, including responsive feeding (RF) and non-responsive feeding (NRF), plays a critical role in shaping the children’s eating behavior [9-11]. RF has been proven to promote healthy growth and development [12], and proposed by the WHO as a core element of nurturing care [1]. On the contrary, non-responsive feeding practices, such as restriction and pressure-to-eat, are likely to result in inappropriate weight gain [13]. A systematic review has indicated that restriction was significantly associated with higher child weight status, while pressure-to-eat was significantly associated with lower child weight status [14]. Moreover, the use of food as a reward or as a source of comfort was associated with child emotional eating and a tendency to overeat in children [14]. It is reported that the prevalence of overweight and obese for Chinese children under six years old in 2015-19 was 6.8 and 3.6%, respectively, accounting for the largest child population with obesity in the world [15]. Therefore, evaluating caregivers’ feeding practices is in great need to prevent obesity in China.

With the advancement of the social economy and the continuous enhancement of people's living standards, an increasing number of Chinese parents began to place significance on their children's early childhood education (ECE), especially in urban China. It is reported that around 70% children aged 0–6 years in first-tier cities and almost 30% in second-tier provincial capitals as well as third- and fourth-tier cities attended ECE institutions in 2020 [16]. In addition, as the children's first teacher, nurturer and protector, primary caregivers play a crucial role in providing nurturing care for infants and young children [17,18]. The most basic experiences in early life come from the nurturing care and protection of parents and families, which have lifelong benefits for health and well-being and enhance the ability to learn and earn [19]. Despite numerous studies on infant feeding, the current status of early childhood feeding in urban ECE institutions remains unknown. This study aims to investigate the complementary feeding practices of children aged 6–23 months in ECE institutions in urban China and explore potential factors influencing children's feeding practices.

## METHODS

### Study design and participants

We conducted a cross-sectional survey with caregivers of children aged 6–23 months in ECE institutions across 31 provinces in China from 1 March to 30 April 2023 to collect data on both children’s and caregivers’ feeding practices.

We collaborated with an ECE institution, Gymboree Play & Music, to recruit participants for the survey, more detail can be found in our previous paper [20]. Primary caregivers of children aged 6–23 months, either enrolled as members of Gymboree Play & Music or as non-members, were invited to participate the survey. We excluded caregivers who did not have WeChat app installed on their smartphone, or had children aged younger than six or older than 23 months old, or refused to participate. Convenience sampling method was used to recruit participants.

#### Survey instrument

Our survey instrument included an IYCF practices questionnaire and a caregivers’ feeding practices scale. The IYCF questionnaire was developed based on adapted WHO Maternal, Newborn and Child Health Household Survey (MNCHHHS) (unpublished, 2009) and “Indicators for assessing infant and young child feeding practices” (WHO & UNICEF) to collect data on children’s complementary feeding practices, and the frequency of seven groups of complementary food during the last month and caregivers’ feeding knowledge and information sources. We translated and adapted the standard survey instruments to make it appropriate for the Chinese context and we have been using the questionnaires in our previous studies during the past decade [21–25].

The caregivers’ feeding practices scale was developed based on our previous scale development study, which consisted of 27 items and six dimensions (pressure to eat, concern about child undereating, emotional feeding or instrumental feeding, prompting and encouragement to eat, restriction, concern about child overeating) [26]. The items were scored on a 5-point Likert-type scale (1 = never, 2 = rarely, 3 = sometimes, 4 = often, 5 = always) [26].

We also collected the information on the frequency of children attending ECE classes during the last month and caregivers’ daily nurturing care time.

#### Data collection

We used the online survey tool Sojump (http://www.sojump.com) to distribute our questionnaire and collect the data [21,22]. We initially sent the quick response (QR) code of our questionnaire to the head of Gymboree Play & Music headquarters, and then the QR code was distributed to the centre directors and teachers. After obtaining caregivers’ oral consent, the teachers at each centre invited caregivers to participate in the survey by scanning the QR code with their own WeChat, and caregivers filled in the questionnaire online. Detailed information of data collection has been reported in our previous paper [20].

#### Outcomes

The primary outcome measure was the prevalence of complementary feeding practices during the last 24 hours by two age groups (6–11 months and 12–23 months), including minimum dietary diversity (MDD), minimum meal frequency (MMF), minimum acceptable diet (MAD), consumption of iron-rich or fortified foods (CIRIFF), consumption of iron-rich foods (CIRF), and continued breastfeeding (CBF) at 12–23 months (**Box 1**).

Box 1 **Complementary feeding practices assessment**

The primary outcomes were children’s complementary feeding practices indicators proposed by the WHO &UNICEF:

1. Minimum dietary diversity 6–23 months (MDD): Percentage of children 6–23 months of age who consumed foods and beverages from at least five out of eight defined food groups during the previous day. The eight food groups used for tabulation of this indicator were: 1) breast milk; 2) grains, roots, tubers and plantains; 3) pulses (beans, peas, lentils), nuts and seeds; 4) dairy products (milk, infant formula, yogurt, cheese); 5) flesh foods (meat, fish, poultry, organ meats); 6) eggs; 7) vitamin A rich fruits and vegetables; and 8) other fruits and vegetables.

2. Minimum meal frequency 6–23 months (MMF): Percentage of children 6–23 months of age who consumed solid, semi-solid or soft foods (but also including milk feeds for non-breastfed children) at least the minimum number of times during the previous day. The minimum number of times was defined as:

• two feedings of solid, semi-solid or soft foods for breastfed infants aged 6–8 months;

• three feedings of solid, semi-solid or soft foods for breastfed children aged 9–23 months;

• four feedings of solid, semi-solid or soft foods or milk feeds for non-breastfed children aged 6–23 months whereby at least one of the four feeds must be a solid, semi-solid or soft feed.

3. Minimum acceptable diet 6–23 months (MAD): Percentage of children 6–23 months of age who consumed a minimum acceptable diet during the previous day. The minimum acceptable diet was defined as:

• for breastfed children: receiving at least the minimum dietary diversity and minimum meal frequency for their age during the previous day;

• for non-breastfed children: receiving at least the minimum dietary diversity and minimum meal frequency for their age during the previous day as well as at least two milk feeds.

4. Consumption of iron-rich or iron-fortified foods (CIRIFF): Percentage of children aged 6–23 months who received iron-rich food or iron fortified food that was specially designed for infants and young children, or that was fortified in the home.

5. Consumption of iron-rich foods (CIRF): Percentage of children aged 6–23 months who received iron-rich or fortified foods.

6. Continued breastfeeding at 12–23 months (CBF): Percentage of children 12–23 months of age who were fed breast milk during the previous day.

The secondary outcomes were the frequency of seven groups of complementary food during the last month and caregivers’ feeding knowledge and practices.

Food frequency for last month: Percentage of children aged 6–23 months who ate seven food groups more than 3 times per week. The seven food groups are: 1) grains, roots, tubers and plantains; 2) vitamin-A rich fruits and vegetables; 3) other fruits and vegetables; 4) flesh foods; 5) eggs; 6) pulses (beans, peas, lentils), nuts and seeds; 7) dairy products (yogurts or cheese).

Caregivers’ feeding knowledge: Percentage of caregivers who know exclusive breastfeeding for up to 6 months; percentage of caregivers who know starting complementary food at 6 months; percentage of caregivers who know continued breastfeeding for 2 years or beyond.

Caregivers’ feeding practices: Percentage of caregivers with mean score ≥3 in six dimensions of feeding practices. Six dimensions are: 1) Pressure to eat; 2) Concern about child undereating; 3) Emotional feeding or instrumental feeding; 4) Prompting and encouragement to eat; 5) Restriction; 6) Concern about child overeating.

The secondary outcome measure was the frequency of seven food groups consumed by children during the last month, with high intake defined as consumption at least three times per week. Additionally, we compared differences in caregivers' feeding knowledge and feeding practices between two age groups.

The third outcome involved comparisons of children's complementary feeding practices during the past 24 hours, food frequency during the last month, as well as caregivers' feeding knowledge and practices between two sub-groups: children who attended ECE classes (attending group) and those who did not attend ECE classes (non-attending group), and children whose primary caregivers’ daily nurturing care time ≥4h (long-NCT group) and those whose primary caregivers’ daily nurturing care time <4h (short-NCT group). We defined children attended ECE classes if they attended ECE classes at least once per week during the last month.

#### Data management and statistical analysis

The sample size calculation for this survey was based on the national prevalence of MAD (43.4%) in 2016–2017 [27]. With a 3% desired level of absolute precision, 5% significant level and 80% power, we calculated that a sample size of 2151 caregivers of children aged 6–23 months would be sufficient. Considering a 20% non-response rate, we supposed to recruit 2689 caregivers in this study.

One member of the research team (XW) was responsible to secure the unique password to Sojump account. After primary caregivers submitted questionnaires, their data were uploaded to the database in the Sojump account. Once data collection was completed, we downloaded the database and converted it into Microsoft Excel sheets for statistical analysis.

We utilised SAS 9.4 software (SAS Institute, Cary, NC) to analyse data. Numbers and proportions were used to describe categorical variables. For the caregivers’ feeding practices scale, the scores of the items in each dimension were summed to obtain a total score of each dimension, and the mean score of each dimensions was calculated. If a dimension’s mean score was higher than 3, we assumed that caregivers had feeding problem in the dimension, and calculate the percentage of caregivers who had such feeding problem. To assess differences between subgroups, we conducted an *X^2^* test for categorical variables. The significance level was set at *P* < 0.05.

#### Ethical approval

The study received ethical approval from the Ethical Committee of the Capital Institute of Pediatrics in Beijing (SHERLL2022041). Gymboree Play & Music teachers first explained the study’s aim and procedure to caregivers. After scanning the QR code, caregivers encountered an online consent form necessitating their agreement and digital signature to proceed with the survey. They were explicitly informed that their involvement in this study would remain confidential. In case of any publication or presentation arising from this study, no personally identifiable information will be disclosed. We also protected our Sojump account with a password and signed a security agreement with Sojump for data protection.

## RESULTS

### Location and sample size

**Figure 1:**
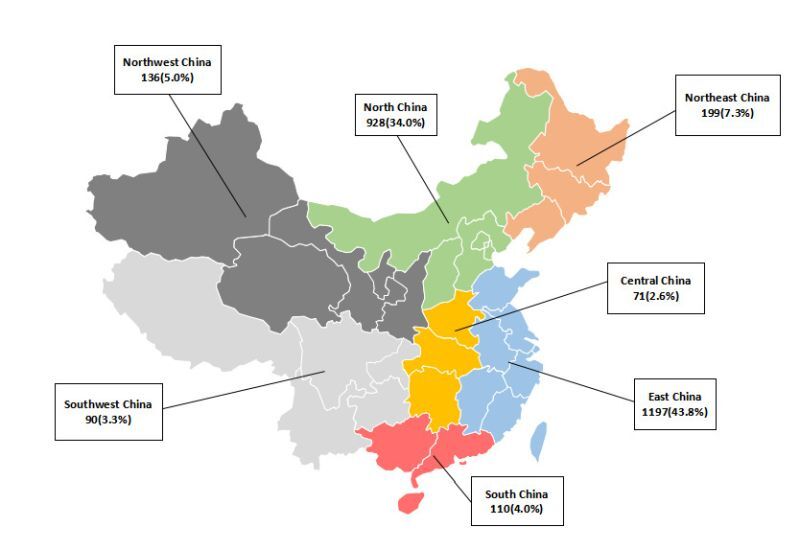
Location map and sample size.

A total of 2731 children aged 6–23 months and their primary caregivers from seven geographical regions in China participated in this survey, with the majority of participants being from East China (43.8%) and North China (34.0%) (**Figure 1**).

### Socio-demographic characteristics

We enrolled 416 children aged 6–11 months and 2315 children aged 12–23 months for analysis. In general, there was an almost 1:1 ratio of males to females, and 93.7% of the children were Han Chinese. During the last month, more than three-fourths of children attended ECE classes, while 62% of primary caregivers spent at least four hours daily providing nurturing care for their children. Among the primary caregivers, mothers accounted for 75.8%, followed by fathers (17.3%), and nearly 90% of them had a college degree or above (**Table 1**).

**Table 1:** Characteristics of surveyed children and their primary caregivers*

Variables	6–11 months, n = 416	12–23 months, n = 2315	Total, n = 2731
**Children**			
Sex			
*Male*	212 (51.0)	1104 (47.7)	1316 (48.2)
*Female*	204 (49.0)	1211 (52.3)	1415 (51.8)
Ethnicity			
*Han*	373 (89.7)	2185 (94.4)	2558 (93.7)
*Ethnic minorities*	43 (10.3)	130 (5.6)	173 (6.3)
Attended ECE classes during the last month	316 (76.0)	1771 (76.5)	2087 (76.4)
**Primary caregivers**			
Relationship			
*Mother*	289 (69.5)	1781 (76.9)	2070 (75.8)
*Father*	81 (19.5)	391 (16.9)	472 (17.3)
*Grandparents*	31 (7.5)	80 (3.5)	111 (4.1)
*Others*	15 (3.6)	63 (2.72)	78 (2.9)
Education			
*Senior high school or below*	80 (19.2)	280 (12.1)	360 (13.2)
*College or above*	336 (80.8)	2035 (87.9)	2371 (86.8)
Primary caregivers’ daily nurturing care time during the last month			
*≥4 h/d*	251 (60.3)	1442 (62.3)	1693 (62.0)
*<4 h/d*	165 (39.7)	873 (37.7)	1038 (38.0)

ECE – early childhood education

*Values presented as n (%) unless specified differently.

#### Practices and knowledge of complementary feeding behaviours

The children’s complementary feeding practices were generally suboptimal, with the prevalence of MDD, MMF and MAD ranging from nearly 40 to 60% during the last 24 hours (**Table 2**). Except for CBF, other five feeding practices indicators in children aged 6–11 months were significantly lower than those in children aged 12–23 months (*P* < 0.0001). The food frequencies during the last month remained not ideal either, with less than 60% of children consuming each single food group at least three times weekly. Apart from dairy products, the consumption frequency of other six food groups in 6–11 months age group was significantly lower compared to that in 12–23 months age group (*P* < 0.05). For feeding knowledge, less than 20% of caregivers believed that children should be continued breastfeeding at two years or above, and less than half caregivers knew that children should be introduced complementary foods at six months. Regarding caregivers’ feeding practices, 82% caregivers could conduct responsive feeding (F4), while the proportion of five non-responsive feeding ranged from 28 to 65%. Additionally, only Restriction (F5) showed statistical significance between two age groups (64.9% for 12–23-month vs. 59.4% for 6–11-month, *P* = 0.0312).

**Table 2:** Complementary feeding practices of surveyed children and caregivers’ feeding knowledge and practices*

Variables	6–11 months, n = 416	12–23 months, n = 2315	Total, n = 2731	*P-*value
**Children’s feeding practices for last 24 h**				
Minimum dietary diversity (MDD)	201 (48.3)	1421 (61.4)	1622 (59.4)	<0.0001
Minimum meal frequency (MMF)	187 (45.0)	1467 (63.4)	1654 (60.6)	<0.0001
Minimum acceptable diet (MAD)	99 (23.8)	972 (42.0)	1071 (39.2	<0.0001
Consumption of iron-rich or iron-fortified foods (CIRIFF)	311 (74.8)	1967 (85.0)	2278 (83.4)	<0.0001
Consumption of iron-rich foods (CIRF)	243 (58.4)	1753 (75.7)	1996 (73.1)	<0.0001
Continued breastfeeding at 12–23 months (CBF)^a^	–	682 (29.5)	682 (29.5)	
**Children’s food frequency for last month**				
Grains, roots, tubers and plantains	149 (35.8)	985 (42.6)	1134 (41.5)	0.0103
Vitamin-A rich fruits and vegetables	179 (43.0)	1233 (53.3)	1412 (51.7)	0.0001
Other fruits and vegetables	200 (48.1)	1381 (59.7)	1581 (57.9)	<0.0001
Flesh foods	131 (31.5)	1053 (45.5)	1184 (43.4)	<0.0001
Eggs	177 (42.6)	1389 (60.0)	1566 (57.3)	<0.0001
Pulses (beans, peats, lentils), nuts and seeds	85 (20.4)	586 (25.3)	671 (24.6)	0.0333
Dairy products (yogurts or cheese)	29 (7.0)	158 (6.8)	187 (6.9)	0.9135
**Caregivers’ feeding knowledge**				
Exclusive breastfeeding up to 6 months	150 (36.1)	852 (36.8)	1002 (36.7)	0.7714
Starting complementary food at 6 months	174 (41.8)	1098 (47.4)	1272 (46.6)	0.0349
Continued breastfeeding for 2 y or beyond	82 (19.7)	389 (16.8)	471 (17.3)	0.1483
**Caregivers’ feeding practices**				
F1 Pressure to eat	160 (38.5)	999 (43.2)	1159 (42.4)	0.0747
F2 Concern about child undereating	122 (29.3)	661 (28.6)	783 (28.7)	0.7479
F3 Emotional feeding or instrumental feeding	116 (27.9)	678 (29.3)	794 (29.1)	0.5619
F4 Prompting and encouragement to eat	340 (81.7)	1900 (82.1)	2240 (82.0)	0.8669
F5 Restriction	247 (59.4)	1502 (64.9)	1749 (64.0)	0.0312
F6 Concern about child overeating	166 (39.9)	926 (40.0)	1092 (40.0)	0.9706

*Values presented as n (%) unless specified differently.

Regarding children’s feeding practices, apart from the CBF, the prevalence of all indicators during the last 24 hours among total children in the attending group was significantly higher compared to that in the non-attending group (MDD = 62.0 vs. 51.1%, *P* < 0.001; MMF = 63.2 vs. 52.2%, *P* < 0.001; MAD = 42.4 vs. 29.0%, *P* < 0.001; CIRIFF = 85.4 vs. 76.9%, *P* < 0.001; CIRF = 76.0 vs. 63.7%, *P* < 0.001), and this pattern was also observed among children aged 12–23 months old. In contrast, the prevalence of CBF in the attending group was significantly lower than that in the non-attending group (28.2 vs. 33.5%, *P* = 0.0194). For high consumption frequency of the seven food groups during the last month, children in the attending group had higher proportions compared to those in the non-attending group (*P* < 0.05), except for pulses (*P* = 0.1660) and dairy products (*P* = 0.7082). Among children aged 12–23 months, only dairy products showed no statistical significance between the two groups, while the proportions of high consumption frequency of other six food groups in the attending group were higher than those in the non-attending group (*P* < 0.05). For caregivers’ feeding knowledge, more caregivers whose children attended ECE classes knew that children should be introduced complementary foods at six months (47.8 for attending group vs. 42.7% for non-attending group, *P* = 0.0241), which revealed a similar pattern among children aged 12–23 months. For caregivers’ feeding practices, those in the attending group demonstrated significantly more responsive feeding (F4) than those in the non-attending group (84.0 vs. 75.8%, *P* < 0.0001). The proportion of F2 and F3 in the attending group was noticeably lower than in the non-attending group (F2 = 26.7 vs. 35.1%, *P* < 0.001; F3 = 27.8 vs. 33.1%, *P* = 0.0105), which also exhibited a similar pattern among children aged 12–23 months. Furthermore, children aged 6–11 months in the attending group demonstrated lower levels of F5 (56.7 vs. 68.0%, *P* = 0.0439) (**Table 3**).

**Table 3:** Comparison of children’s complementary feeding practices and caregivers’ feeding knowledge and practices between children who attended (attending group) and non-attended ECE classes (non-attending group)*

Variables	6–11 months			12–23 moonths			Total		
	**Attending group, n = 316**	**Non-attending group, n = 100**	***P-*value**	**Attending group, n = 1771**	**Non-attending group, n = 544**	***P-*value**	**Attending group, n = 2087**	**Non-attending group, n = 644**	***P-*value**
**Children’s feeding practices for last 24 h**									
Minimum dietary diversity (MDD)	155 (49.1)	46 (46.0)	0.5947	1138 (64.3)	283 (52.0)	<0.0001	1293(62.0)	329 (51.1)	<0.0001
Minimum meal frequency (MMF)	142 (44.9)	45 (45.0)	0.9912	1176 (66.4)	291 (53.5)	<0.0001	1318 (63.2)	336 (52.2)	<0.0001
Minimum acceptable diet (MAD)	79 (25.0)	20 (20.0)	0.3062	805 (45.5)	167 (30.7)	<0.0001	884 (42.4)	187 (29.0)	<0.0001
Consumption of iron-rich or iron-fortified foods (CIRIFF)	238 (75.3)	73 (73.0)	0.6421	1545 (87.2)	422 (77.6)	<0.0001	1783 (85.4)	495 (76.9)	<0.0001
Consumption of iron-rich foods (CIRF)	189 (59.8)	54 (54.0)	0.3042	1397 (78.9)	356 (65.4)	<0.0001	1586 (76.0)	410 (63.7)	<0.0001
Continued breastfeeding at 12–23 months (CBF)†	–	–	–	500 (28.2)	182 (33.5)	0.0194	500 (28.2)	182 (33.5)	0.0194
**Children’s food frequency for last month**									
Grains, roots, tubers and plantains	107 (33.9)	42 (42.0)	0.1390	784 (44.3)	201 (37.0)	0.0025	891 (42.7)	243 (37.7)	0.0256
Vitamin-A rich fruits and vegetables	135 (42.7)	44 (44.0)	0.8219	985 (55.6)	248 (45.6)	<0.0001	1120 (53.7)	292 (45.3)	0.0002
Other fruits and vegetables	150 (47.5)	50 (50.0)	0.6588	1098 (62.0)	283 (52.0)	<0.0001	1248 (59.8)	333 (51.7)	0.0003
Flesh foods	98 (31.0)	33 (33.0)	0.7092	860 (48.6)	193 (35.5)	<0.0001	958 (45.9)	226 (35.1)	<0.0001
Eggs	142 (44.9)	35 (35.0)	0.0798	1096 (61.9)	293 (53.9)	0.0008	1238 (59.3)	328 (50.9)	0.0002
Pulses (beans, peats, lentils), nuts and seeds	57 (18.0)	28 (28.0)	0.0313	469 (26.5)	117 (21.5)	0.0196	526 (25.2)	145 (22.5)	0.1660
Dairy products (yogurts or cheese)	23 (7.3)	6 (6.0)	0.6617	122 (6.9)	36 (6.6)	0.8264	145 (7.0)	42 (6.5)	0.7082
**Caregivers feeding knowledge**									
Exclusive breastfeeding up to 6 months	117 (37.0)	33 (33.0)	0.4650	669 (37.8)	183 (33.6)	0.0802	786(37.7)	216 (33.5)	0.0578
Starting complementary food at 6 months	133 (42.1)	41 (41.0)	0.8475	864 (48.8)	234 (43.0)	0.0184	997 (47.8)	275 (42.7)	0.0241
Continued breastfeeding for 2 y or beyond	62 (19.6)	20 (20.0)	0.9337	293 (16.5)	96 (17.7)	0.5474	355 (17.0)	116 (18.0)	0.5561
**Caregivers’ feeding practices**									
F1 Pressure to eat	122 (38.6)	38 (38.0)	0.9133	755 (42.6)	244 (44.9)	0.3602	877 (42.0)	282 (23.6)	0.4278
F2 Concern about child undereating	88 (27.9)	34 (34.0)	0.2389	469 (26.5)	192 (35.3)	<0.0001	557 (26.7)	226 (35.1)	<0.0001
F3 Emotional feeding or instrumental feeding	89 (28.2)	27 (27.0)	0.8209	492 (27.8)	186 (34.2)	0.0041	581 (27.8)	213 (33.1)	0.0105
F4 Prompting and encouragement to eat	259 (82.0)	81 (81.0)	0.8282	1493 (84.3)	407 (74.8)	<0.0001	1752 (84.0)	488 (75.8)	<0.0001
F5 Restriction	179 (56.7)	68 (68.0)	0.0439	1163 (65.7)	339 (62.3)	0.1519	1342 (64.3)	407 (63.2)	0.6098
F6 Concern about child overeating	125 (39.6)	41 (41.0)	0.7973	700 (39.5)	226 (41.5)	0.4006	825 (39.5)	267 (41.5)	0.3823

ECE – early childhood education

*Values presented as n (%) unless specified differently.

†Denominator for this indicator is children aged 12–23 months.

The prevalence of MMF and MAD in the long-NCT group was significant higher than that in the short-NCT group among children aged 12–23 months (MMF = 66.2 vs. 58.8%, *P* = 0.0003; MAD = 44.2 vs. 38.3%, *P* = 0.0047), and the prevalence of CBF was significantly higher in the long-NCT group (31.2 vs. 26.6%, *P* = 0.0178). However, all indicators of feeding practices during the last 24 hours showed no differences between the two groups among children aged 6–11 months. For high consumption food frequency during the last month, the long-NCT group exhibited higher consumption in four out of seven food groups among total children. In the 12–23 months group, three out of the seven food groups showed significant differences, with higher consumption in the long-NCT group than that in the short-NCT group (other fruits and vegetables = 61.8 vs. 56.1%, *P* = 0.0071; eggs = 62.2 vs. 56.4%, *P* = 0.0054; pulses, nuts, and seeds = 26.8 vs. 22.9%, *P* = 0.0385). Among children aged 6–11 months, only fruits and vegetables was significantly higher in the long-NCT group (52.6 vs. 41.2%, *P* = 0.0231). Furthermore, caregivers who spent longer time on daily nurturing care demonstrated higher knowledge level on starting complementary foods at six months (49.2 vs. 44.6%, *P* = 0.0314) and continued breastfeeding (18.2 vs. 14.4%, *P* = 0.0176) among children aged 12–23 months. In 6–11 months age group, more caregivers in the long-NCT group knew that children should be continued breastfed up to two years or above compared to those in the short-NCT group (23.1 vs. 14.6%, *P* = 0.0318). Regarding caregivers' feeding practices, caregivers in the long-NCT group exhibited better responsive feeding (F4) among total children (84.8 vs. 77.5%, *P* < 0.0001). Except for F5 (66.0 vs. 60.9%, *P* = 0.0071), fewer caregivers in the long-NCT group demonstrated negative feeding practices on F1 (40.8 vs. 45.1%, *P* = 0.0284) and F2 (27.3 vs. 30.9%, *P* = 0.0414) compared to their counterparts (**Table 4**).

**Table 4:** Comparison of children’s complementary feeding practices and caregivers’ feeding knowledge and practices between children with nurturing care time ≥4 h (long-NCT group) and those with daily nurturing care time <4 h (short-NCT group)*

Variables	6–11 months			12–23 months			Total		
	**≥4h, n = 251**	**<4h, n = 165**	***P-*value**	**≥4h, n = 1442**	**<4h, n = 873**	***P-*value**	**≥4h, n = 1693**	**<4h, n = 1038**	***P-*value**
**Children’s feeding practices for last 24 h**									
Minimum dietary diversity (MDD)	128 (51.0)	73 (44.2)	0.1775	898 (62.3)	523 (59.9)	0.2571	1026 (60.6)	596 (57.4)	0.1000
Minimum meal frequency (MMF)	114 (45.4)	73 (44.2)	0.8135	954 (66.2)	513 (58.8)	0.0003	1068 (63.1)	586 (56.5)	0.0006
Minimum acceptable diet (MAD)	61 (24.3)	38 (23.0)	0.7656	638 (44.2)	334 (38.3)	0.0047	699 (41.3)	372 (35.8)	0.0046
Consumption of iron-rich or iron-fortified foods (CIRIFF)	190 (75.7)	121 (73.3)	0.5872	1224 (84.9)	743 (85.1)	0.8824	1414 (83.5)	864 (83.2)	0.8468
Consumption of iron-rich foods (CIRF)	150 (59.8)	93 (56.4)	0.4916	1102 (76.4)	651 (74.6)	0.3140	1252 (74.0)	744 (71.7)	0.1931
Continued breastfeeding at 12–23 months (CBF)†	–	–	–	450 (31.2)	232 (26.6)	0.0178	450 (31.2)	232 (26.6)	0.0178
**Children’s food frequency for last month**									
Grains, roots, tubers and plantains	90 (35.9)	59 (35.8)	0.9836	625 (43.3)	360 (41.2)	0.3207	715 (42.2)	419 (40.4)	0.3366
vitamin-A rich fruits and vegetables	114 (45.4)	65 (39.4)	0.2247	787 (54.6)	446 (51.1)	0.1030	901 (53.2)	511 (49.2)	0.0428
Other fruits and vegetables	132 (52.6)	68 (41.2)	0.0231	891 (61.8)	490 (56.1)	0.0071	1023 (60.4)	558 (53.8)	0.0006
Flesh foods	83 (33.1)	48 (29.1)	0.3929	678 (47.0)	375 (43.0)	0.0571	761 (45.0)	423 (40.8)	0.0316
Eggs	113 (45.0)	64 (38.8)	0.2085	897 (62.2)	492 (56.4)	0.0054	1010 (59.7)	556 (53.6)	0.0018
Pulses (beans, peats, lentils), nuts and seeds	51 (20.3)	34 (20.6)	0.9433	386 (26.8)	200 (22.9)	0.0385	437 (25.8)	234 (22.5)	0.0541
Dairy products (yogurts or cheese)	17 (6.8)	12 (7.3)	0.8447	102 (7.1)	56 (6.4)	0.5424	119 (7.0)	68 (6.6)	0.6312
**Caregivers’ feeding knowledge**									
Exclusive breastfeeding up to 6 months	93 (37.1)	57 (34.6)	0.6025	545 (37.8)	307 (35.2)	0.2037	638 (37.7)	364 (35.1)	0.1684
Starting complementary food at 6 months	107 (42.6)	67 (40.6)	0.6823	709 (49.2)	389 (44.6)	0.0314	816 (48.2)	456 (43.9)	0.0300
Continued breastfeeding for 2 years or beyond	58 (23.1)	24 (14.6)	0.0318	263 (18.2)	126 (14.4)	0.0176	321 (19.0)	150 (14.5)	0.0025
**Caregivers’ feeding practices**									
F1 Pressure to eat	89 (35.5)	71 (43.0)	0.1204	602 (41.8)	397 (45.5)	0.0792	691 (40.8)	468 (45.1)	0.0284
F2 Concern about child undereating	66 (26.3)	56 (33.9)	0.0939	396 (27.5)	265 (30.4)	0.1352	462 (27.3)	321 (30.9)	0.0414
F3 Emotional feeding or instrumental feeding	71 (28.3)	45 (27.3)	0.8215	409 (28.4)	269 (30.8)	0.2093	480 (28.4)	314 (30.3)	0.2889
F4 Prompting and encouragement to eat	211 (84.1)	129 (78.2)	0.1288	1225 (85.0)	675 (77.3)	<.0001	1436 (84.8)	804 (77.5)	<0.0001
F5 Restriction	148 (59.0)	99 (60.0)	0.8333	969 (67.2)	533 (61.1)	0.0027	1117 (66.0)	632 (60.9)	0.0071
F6 Concern about child overeating	97 (38.7)	69 (41.8)	0.5180	571 (39.6)	355 (40.7)	0.6117	668 (39.5)	424 (40.9)	0.4713

NCT – nurturing care time

*Values presented as n (%) unless specified differently.

†Denominator for this indicator is children aged 12–23 months.

**Figure 2:**
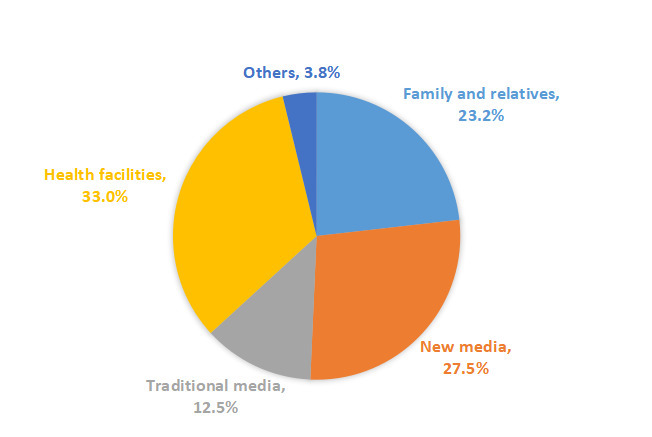
Source of information on complementary feeding knowledge.

Among the surveyed caregivers, 1539 (56.4%) of them reported obtaining feeding knowledge from various sources. Health facilities were identified as the most common source, accounting for more than 30%, followed by new media (27.5%), family and relatives (23.2%), and traditional media including newspapers, broadcast, and TV (12.5%) (**Figure 2**).

## DISCUSSION

This cross-sectional study aimed to evaluate the status of complementary feeding practices among children aged 6–23 months in ECE institutions in urban China. The key findings of this study indicate that feeding practices of both children and caregivers were suboptimal. Moreover, children who attended ECE classes and whose primary caregivers’ nurturing care time was ≥4 hours/d demonstrated higher adherence to complementary feeding practices than their counterparts.

Our findings indicated that 59.4, 60.6, and 39.2% of children in ECE institutions met MDD, MMF, and MAD respectively, which were aligned closely with China Nutrition and Health Surveillance data in 2016–2017 (60.6, 72.4, and 43.4% respectively) [8], but higher than global level in 2017 (28.2, 50.3, and 15.9%, respectively) [7]. For children age 6–23 months, feeding a variety of foods will help them get all their nutritional needs, and lacking of dietary diversity can increase the risk of micronutrient deficiencies, which could be detrimental to physical and cognitive development [28]. However, around 40% children in our study did not meet MDD, which needs be further improved. Additionally, our results found that only 17.3% caregivers believed children should be continued breastfeeding at two years or above, and 29.5% children aged 12–23 months were continued breastfed, in line with our previous study conducted in rural Qinghai, China [24]. During the second year of life, breast milk continues to provide energy, micronutrient and immune protection to the child [1], therefore, much efforts is required to promote continued breastfeeding in China.

In addition, non-responsive feeding practices of caregivers were observed in our study, such as restriction (64%), pressure to eat (42.4%), emotional feeding or instrumental feeding (29.1%). The inappropriate feeding behaviour is primarily attributed to parental control, coercion, or neglect in feeding practices, as well as a failure to discern the child's hunger and satiety cues [29]. These improper feeding practices of caregivers may give undue pressure on children, impeding the development of their self-regulation abilities in relation to eating habits, and potentially increasing their susceptibility to obesity later in life [30]. Evidence suggests restrictive feeding practices are associated with increased weight gain and higher weight status [31]. Findings in our study are consistent with previous studies indicating a tendency among parents towards reliance on control-oriented approaches, while utilising fewer structure-based strategies such as family meal settings [32,33]. Therefore, it is necessary to provide responsive feeding guidance to teach caregivers to recognise and respond appropriately to children’s hunger and satiety cues, which can lead to ‘normal weight gain and/or ‘normal’ weight status in children under 2 years old [31].

Our study also indicated that children who attended ECE classes demonstrated better complementary feeding practices in 12–23 months age group. However, it is noteworthy that the majority of ECE institutions in China lack relevant information or service on IYCF. The possible reason is that caregivers with children attending ECE classes are more inclined to pay attention to complementary feeding and access relative information via alternative channels compared to those not attending. Researches have demonstrated that caregivers with a higher understanding of ECE are more likely to seek knowledge from diverse sources [34,35], and enrol their children in ECE classes [35]. Given the increasing emphasis placed by both the Chinese government and parents on early childhood development for children under three years old, it is crucial to leverage the ECE institutions, as well as other delivery channels, to disseminate recommended IYCF information, thereby improving feeding practices in China. Future studies are needed to test the effectiveness of this potential delivery channel in China.

Our findings highlighted the importance of caregivers’ nurturing care time on IYCF. Children whose primary caregivers spent ≥4 h/d on nurturing care (long-NCT group) exhibited significantly better feeding practices compared to those in short-NCT group. Given that the family serves as the main environment for infants and young children, fostering a positive home environment can promote feeding practices and influence children’s eating habits [36]. Caregivers, acting as their most influential role models, have a pivotal impact on shaping children's eating behavior through their words and action [33]. Caregivers who spend more time on nurturing care are likely to provide stress-free encouragement and structured mealtime routines, thereby contributing to improve feeding outcomes [34,35].

Our study also has some limitations. First, this study investigated the status of complementary feeding of children in ECE institutions in Chinese cities. We used convenient sample rather than random sample, and collected data through self-reported questionnaires, both of which may cause bias. However, the results of our study were aligned with the national surveys. Second, our study was a unicentrical study as we only collaborated with a single ECE institution, Gymboree Play & Music, which may lead to selection bias; therefore, caution should be exercised when generalising the findings to other ECE settings. Third, we used the online survey tool to collect data, which may introduce potential self-selection bias.

## CONCLUSIONS

In summary, the complementary feeding practices of children aged 6–23 months in ECE institutes in urban China was not optimal, and non-responsive feeding practices remained common among caregivers. The attendance of ECE classes and the caregivers’ daily nurturing time could be beneficial in ensuring children to adhere to complementary feeding guidelines and recommendations, particularly for children aged 12–23 months. Multiple channels, including health facilities, ECE institutions and new media, should be utilized to effectively promote IYCF practices in China.
